# COVID-19-Induced Hypoxia With Accompanying Syncope Event and Traumatic Injury

**DOI:** 10.7759/cureus.14602

**Published:** 2021-04-21

**Authors:** Christ Ordookhanian, Ryan F Amidon, Sean W Kaloostian, Talia Vartanian, Paul Kaloostian

**Affiliations:** 1 Medicine, University of California Riverside School of Medicine, Riverside, USA; 2 Medicine, Medical College of Wisconsin, Milwaukee, USA; 3 Neurological Surgery, Haider Spine Center, Riverside, USA; 4 Physical Medicine and Rehabilitation, University of Southern California, Los Angeles, USA; 5 Medicine, Riverside Community Hospital, Riverside, USA

**Keywords:** covid-19, coronavirus disease, brain hypoxia, acute trauma care, anti-coagulation, syncope, coronavirus pandemic, d-dimer, acute respiratory distress syndrome (ards)

## Abstract

The Centers for Disease Control and Prevention identified the first case of the novel coronavirus disease 2019 (COVID-19) on January 21, 2020 in the United States. Since its arrival, the virus has caused widespread havoc on the nation as a whole as well as on all individuals. The coronavirus family is not new to the field of medicine. In fact, the viral pathogenicity dates back to the early 1960s, with more information on the respiratory preference and the ability to cause acute respiratory pathology coming later in 2002. The novel coronavirus, severe acute respiratory syndrome coronavirus 2, causes a disease commonly referred to as COVID-19, which has a well documented course of severe respiratory pathology along with interesting systemic consequences that often complicate the clinical picture. This case presents an otherwise healthy young 35-year-old male who contracted the novel coronavirus, leading to multi-organ hypoxia and triggering a syncopal episode which resulted in physical trauma to the head leading to a minor subarachnoid hemorrhage.

## Introduction

Since the arrival of the novel coronavirus disease 2019 (COVID-19) to American shores in early January 2020, the virus has demonstrated its severe pathogenicity and ability to cause concomitant systemic pathology that paints a complicated clinical picture. As per the Centers for Disease Control and Prevention, patients often experience a wide range of symptoms, with the most common being fever with chills, cough, shortness of breath, difficulty breathing, fatigue, and loss of taste and smell [1]. As the virus progressed, it became clear that the coronavirus primarily affected the lungs, with a study highlighting the right lung being preferred for initial infection over the left, but with an overwhelming consensus that a vast majority of the time morbidity and mortality were tightly linked with lung injury [2]. It was quickly realized that aerosolized transmission of the virus is what led to a remarkably higher rate of transmission, with the risk behind high-concentration inhalation inoculation being the driving factor behind the social distancing campaign. Upon entrance of the virus into the airway, the virus seeds into the alveoli of the lungs, forming a linkage between angiotensin-converting enzyme two of the type two pneumocytes via the virus’s spike protein [3]. The resulting cytokine storm of interleukin one (IL-1), six (IL-6), and tumor necrosis factor alpha (TNF alpha) is vastly responsible for both the clinical symptoms and the pathophysiology of pulmonary injury due to interstitial inflammation, pulmonary edema, acute bronchitis, and even acute respiratory distress syndrome (ARDS) [3,4]. The clinical picture is not new to medicine, often termed “cold-like” or “flu-like” symptoms, leading to the public’s misunderstanding and understating of the pathogenicity of the virus. The cytokine storm, which is responsible for the symptoms, is congruent with systemic inflammation with more symptoms raising concern to the pulmonary system due to nearly 67.8% of the surveyed patients reporting cough and 18.6% reporting shortness of breath, which is substantially higher than the yearly influenza virus (less than 5% and 11% for both symptoms, respectively) [3-5]. With the virus predominating in the lungs, systemic inflammation and the resulting wide-spread pathology was also of key concern as studies found the novel coronavirus brought with it remarkable systemic complications such as acute coronary syndrome, heart failure, myocarditis, cardiac arrhythmias, acute kidney injury, anemia, leukocytosis, hypersensitivity reactions, hypercoagulable states, increased pro-coagulation factors, abdominal discomfort with resulting symptomology, and even associated psychiatric symptoms such as delirium, psychosis, visual impairment, smell and taste alterations, and depression [3,4]. In this case, we highlight the systemic consequences of the coronavirus infection, which led to a stepwise decline of health in our patient. A healthy, relatively young adult male contracted the novel coronavirus, later experiencing respiratory distress leading to hypoxia which resulted in a loss of consciousness that led to a traumatic brain injury (TBI) after making contact with an unknown foreign object within his home. This sequence of events also led to the discovery of the systemic effects of the novel coronavirus as laboratory testing revealed acute kidney changes and elevated pro-coagulable factors.

## Case presentation

A 35-year-old male respiratory therapist with high occupational exposure risk to COVID-19 presented to the emergency department after an unwitnessed reported fall by roommates while at home. The roommates reported a “thump” and rushed to find the patient unconscious in close proximity to a hard surface with visible skin trauma and bleeding in the bathroom. The patient had no prior history of seizures and no family history of seizures or seizure disorders; additionally, the roommates did not report observing an active seizure upon arrival to the patient. Paramedics reported a Glasgow Coma Score (GCS) of 3 at the scene with minimal improvement during transport. A roommate reported that the patient experienced shortness of breath prior to the syncopal episode. Emergency department evaluation revealed a consistent and unimproved GCS of 3 and the patient was subsequently intubated. A 12-lead electrocardiogram was completed, showing regular rate, rhythm, normal axis, no present arrhythmia, and all intervals and QRS complex within normal limits. No ST changes were noted and T-waves were upright in all leads. The patient’s roommates, who were good friends, reported no history of drug use and the patient denied drug use; additionally, the urine drug screen was negative for drug use. The patient’s vitals remained stable throughout the hospital encounter. Most remarkably, the low oxygen saturation observed was consistent with severe cases of COVID-19 (Table 1).

**Table 1:** Initial complete metabolic panel with complete blood count and coagulation studies.

Laboratory test	Emergency department arrival	12^th^ hour in hospital	24^th^ hour in hospital
Sodium	141 mEq/L	142 mEq/L	140 mEq/L
Potassium	3.7 mEq/L	3.9 mEq/L	4.1 mEq/L
Chloride	109 mEq/L	109 mEq/L	112 mEq/L
Carbon dioxide	25 mEq/L	24 mEq/L	22 mEq/L
Anion gap	7	5	6
Blood urea nitrogen	20 mg/dL	23 mg/dL	27 mg/dL
Creatinine	0.870 mg/dL	0.920 mg/dL	0.950 mg/dL
Estimated glomerular filtration rate	96 mL/min/1.73 m^2^	90 mL/min/1.73m^2^	87 mL/min/1.73m^2^
Blood urea nitrogen/creatinine ratio	22.9	25.0	28.4
Glucose	87 mg/dL	89 mg/dL	91 mg/dL
Calcium	8.5 mg/dL	8.5 mg/dL	8.5 mg/dL
Phosphate	2.9 mg/dL	2.9 mg/dL	2.5 mg/dL
Magnesium	1.9 mg/dL	2.5 mg/dL	2.2 mg/dL
Total bilirubin	0.8 mg/dL	0.8 mg/dL	0.7 mg/dL
Aspartate transaminase	37 mU/mL	48 mU/mL	52 mU/mL
Alanine aminotransferase	84 mU/mL	88 mU/mL	98 mU/mL
Prothrombin time	13.5 s		
Partial thromboplastin time	21.9 s		
International normalized ratio	0.884		
D-dimer	4550 ng/mL	4660 ng/mL	4284 ng/mL
White blood cell count	14.7 × 10^9^/L	17.4 × 10^9^/L	17.8 × 10^9^/L
Red blood cell count	5.54 × 10^12^/L	5.27 × 10^12^/L	5.40 × 10^12^/L
Hemoglobin	16.8 g/dL	16.3 g/dL	16.4 g/dL
Hematocrit	50%	49.5%	50.6%
Red cell distribution width	14.9%	15%	15.6%
Platelet count	107 × 10^9^/L	105 × 10^9^/L	110 × 10^9^/L
% Neutrophils	78.6	82.9	92.0
% Lymphocytes	9.6	8.1	4.6

**Figure 1:**
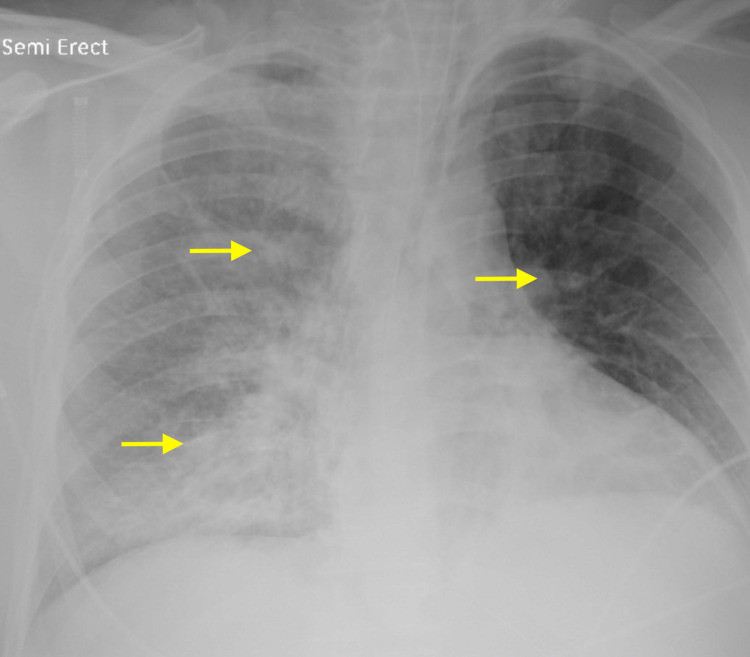
Chest X-ray reveals lung changes characteristic of COVID-19. COVID-19: coronavirus disease 2019

Within the emergency department, a chest radiography was obtained revealing white lung fields, worse on the right side, with multifocal regions of high-density consolidations and vast inflammation (Figure 1).

**Figure 2:**
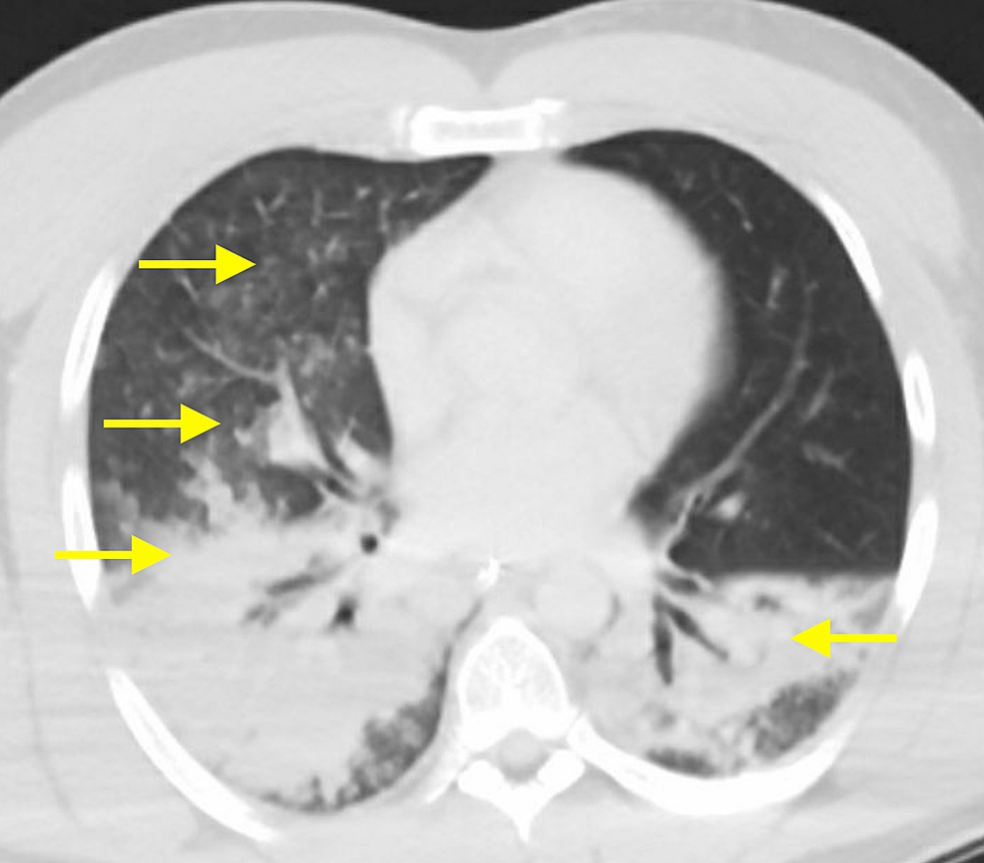
Chest CT scan reveals lung changes characteristic of COVID-19. CT: computed tomography; COVID-19: coronavirus disease 2019

Additionally, a chest computed tomography (CT) scan was completed revealing consolidations throughout the lungs with a multifocal pattern, in line with novel coronavirus findings and the patient’s clinical presentation (Figure 2).

**Figure 3:**
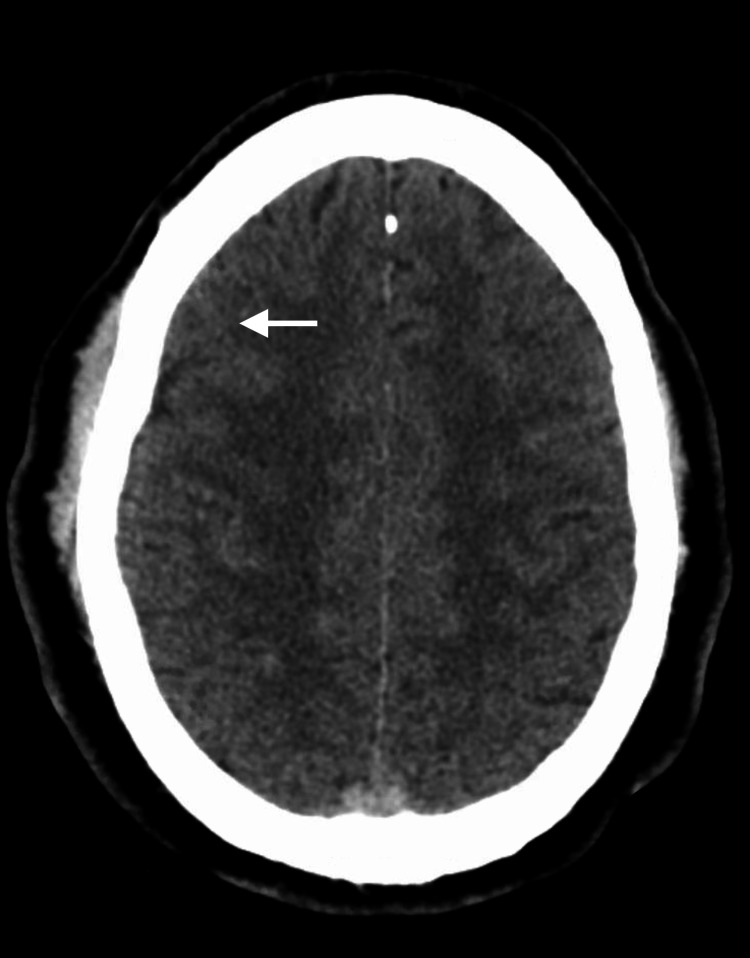
Head CT scan reveals small right parietal subarachnoid hemorrhage. CT: computed tomography

A CT scan of the patient’s head revealed a small area of right parietal traumatic subarachnoid hemorrhage (tSAH), also reported by radiology (Figure 3).

The patient was admitted to the neurological intensive care unit and started on 500 mg of levetiracetam intravenously administered twice a day for seizure prophylaxis. The patient’s labs were notable for being positive for COVID-19 via a reverse transcription polymerase chain reaction test, a hypercoagulable state with depressed international normalized ratio (INR) and elevated D-dimer levels, as well as abnormal complete blood count with abnormal differentials. The patient’s complete blood count was remarkable for leukocytosis with marked neutrophilia, increased red cell distribution width, and mild thrombocytopenia (Table 2).

**Table 2:** Initial patient vital signs.

Vital	Emergency department arrival	12^th^ hour in hospital	24^th^ hour in hospital
Temperature	98.7 ℉	99 ℉	98.5 ℉
Pulse rate	75 beats/min	69 beats/min	68 beats/min
Respiratory rate	18 breaths/min	18 breaths/min	20 breaths/min
Blood pressure	131/80 mmHg	143/78 mmHg	139/90 mmHg
Pulse oximetry (SpO_2_)	86%	85%	86%

**Figure 4:**
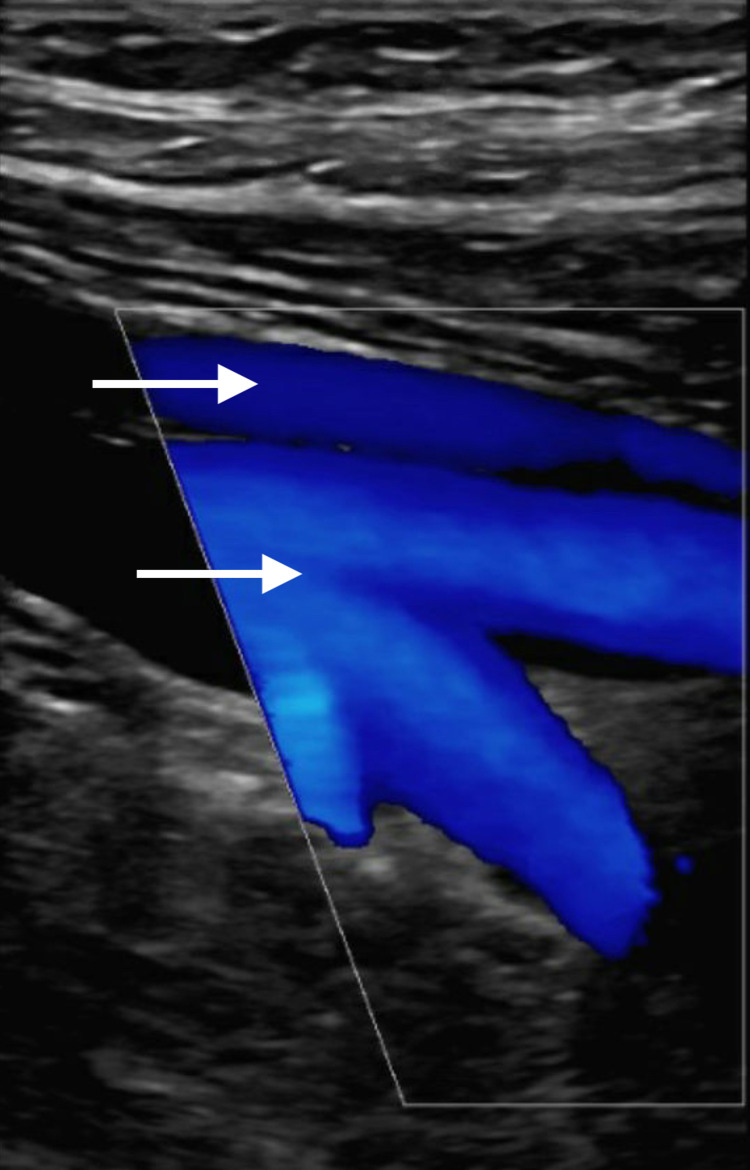
Ultrasonography of right proximal superficial and deep femoral vein reveals normal vasculature.

Given the patient’s concerning D-dimer, prothrombin time (PT), and INR figures, ultrasonography of the right proximal superficial and deep femoral vein was completed to rule out the possibility of a deep vein thrombosis given the patient’s right leg clinical symptoms of edema, warmth, and erythematous appearance (Figure 4).

Ultrasonography revealed normal femoral vein vasculature. The Wells Pretest Probability model for assessing the risk of the patient having a pulmonary embolism or a deep venous thrombosis was enacted revealing moderate risk leading to subsequent CT angiogram with radiologist read of “normal pulmonary vasculature.” The patient received 6 mg of dexamethasone for 10 days, then tapered from 0.5 mg to 0 mg over the course of an additional 12 days, to control lung injury from COVID-19. Anticoagulation therapy was indicated from elevated D-dimer levels and aspirin was administered with a 325 mg loading dose followed by 81 mg per day until discharge. Over the ensuing few days, the patient’s neurological status improved and a repeat CT scan of the head did not show clear hemorrhage any further. The patient was able to be extubated and returned to his baseline neurological status prior to discharge eight days post-admission.

## Discussion

This case report highlights a case of blunt head trauma and TBI with tSAH of the right parietal lobe and diffuse axonal injury (DAI). An initial GCS of 3 indicated severe TBI which is characterized by a considerably high mortality rate. One study found blunt trauma to account for 63% of head injuries, and blunt head trauma coupled with a GCS of 3 was associated with a 65% mortality rate. Only 10% of the patients achieved good functional outcomes at discharge [6]. TBI-induced tSAH and DAI are understandably associated with lower GCS. Further, one European study found tSAH to be heavily indicative of poor outcomes, with only 15% of TBI patients with tSAH achieving favorable recovery compared to 41% of TBI patients without tSAH [7]. DAI, frequently associated with severe blunt head trauma, typically affects white matter tracts and clinical manifestations include headaches, dizziness, nausea, vomiting, fatigue, coma, and/or dysautonomia [8]. Maxwell and Graham found roughly 10% of patients with TBI to have DAI, which was associated with a mortality rate of about 25% [8]. Statistically, our patient’s odds of good recovery were rather poor.

Our patient’s history and laboratory results painted a more complex clinical picture. Respiratory distress, characterized by shortness of breath from COVID-19, may have led to a loss of consciousness preceding the TBI. Glomerular filtration rate (GFR) was slightly reduced and the blood urea nitrogen (BUN)/creatinine ratio was elevated, with a negative drug screen. BUN/creatinine ratio is increased by steroids such as dexamethasone, which the patient received to control inflammation-mediated lung injury, and the GFR value was on the verge of normal, indicating that these values were within reason. Serum liver enzymes (aspartate aminotransferase, AST, and alanine aminotransferase, ALT) were elevated as well; however, this alone does not signify liver damage as AST and ALT increases are common post-TBI or hemorrhage [9]. While serum liver enzyme elevation is occasionally attributed to anticonvulsant medication, this is very unlikely with levetiracetam. The red cell distribution width (RDW) value was similarly increased. Normally, this would indicate a potential nutrient deficiency; however, RDW is also a prognostic factor for severe COVID-19 [10]. RDW levels, as well as reticulocyte (RET) levels, were significantly higher in COVID-19 patients with poor outcomes than in those with favorable outcomes. Wang et al. postulated that elevated RET levels could explain why RDW levels were elevated in COVID-19 patients with poor outcomes since RET and RDW values are proportional. The low lymphocyte and high neutrophil counts observed in our patient were characteristic of hospitalized COVID-19 patients [11].

D-dimer levels were elevated, indicating increased formation and breakdown of fibrin clots. As fibrin clots dissolve, fibrin degenerates into D-dimers. Elevated D-dimer levels increase the risk of pulmonary embolism and one study found that COVID-19 patients with high D-dimer levels experienced a higher mortality incidence than those with low levels [12]. Anticoagulation therapy is therefore an important consideration for COVID-19 patients with high D-dimer counts. The PT test result, normalized with the INR, was slightly elevated, indicating that blood was clotting at a slower rate. The partial thromboplastin time test result was marginally elevated, which would indicate faster blood clotting; however, this value was on the verge of normal and the INR value takes clinical precedence. Slowed clotting is explained by the administration of aspirin in response to elevated D-dimer levels. Additionally, platelet count was reduced. When primary hemostasis began via platelet plugs, platelets were consumed, lowering their count. Secondary hemostasis is characterized by stronger fibrin clots.

Three unique pathophysiological aspects of COVID-19 infection include hypoxemic respiratory failure, cytokine storm, and a hypercoagulable state. The virus can directly damage pneumocytes and/or induce reduced surfactant levels, leading to atelectasis (lung collapse) in cases of COVID-19-induced respiratory distress [13]. COVID-19-associated lung injury may involve alveolar damage, edema, inflammatory cell infiltration, microvascular thrombosis, microvascular damage, and hemorrhage [14]. The resulting diffuse lung damage can progress to respiratory failure and death, so dexamethasone is commonly administered to reduce progression [15]. The systemic hypoxia observed in our patient was due to either ARDS or reduced blood circulation coupled with pulmonary vasoconstriction. One multicenter prospective cohort study found that COVID-19 patients with ARDS encountered double the incidence of thrombotic complications when compared to COVID-19-negative patients afflicted by ARDS [16]. This further demonstrates the importance of anticoagulation therapy in patients with COVID-19-induced ARDS. Cytokine storm, which is triggered by neutrophil and monocyte infiltration of lung tissue, is characterized by elevated IL-1, IL-6, TNF alpha, ferritin, and C-reactive protein [3,13,15]. Upregulated cytokines are known to activate the hypothalamic-pituitary-adrenocortical (HPA) axis. A hyperactivated HPA axis coexisting with lung injury results in elevated neutrophil and reduced lymphocyte counts characteristic in COVID-19 patients, as well as eventual immunosuppression. It is also postulated that cytokine upregulation may disrupt brain function, influencing changes in consciousness [14]. Finally, the prothrombotic state established by COVID-19 infection is marked by elevated fibrin and fibrinogen.

Interestingly, COVID-19 outcomes disproportionately favor the female sex over males, although both sexes experience an equal risk of infection. In a review of over 3,000,000 cases in 46 countries, male COVID-19 patients were admitted to the ICU at three times the rate of females with COVID-19, with higher mortality rates [17]. Peckham et al. suggested that this may be largely due to differences in the adaptive and humoral immune system responses between the sexes, among other factors.

Our patient’s spell of unconsciousness was likely a result of COVID-19-induced respiratory distress and systemic hypoxia reducing the oxygen supply to the brain. The subsequent blunt head trauma and consequential presence of tSAH and DAI, with a GCS of 3, significantly increased the severity of our patient’s condition. The prevention of secondary complications, such as seizure, hypoxia, hypotension, edema, and intracranial hypertension was prioritized, playing a key role in our patient’s successful recovery [8]. The patient received levetiracetam for seizure prophylaxis, dexamethasone for hypoxia, and aspirin for anticoagulation therapy. Fortunately, the patient’s neurological condition improved as the tSAH was relieved, finally returning to baseline eight days post-admission.

## Conclusions

During trying times of the novel coronavirus disease, the medical profession has grappled with the rapidly evolving severity of the virus. During the initial months, severe pathology was seen across hospital systems worldwide, each presenting a unique learning opportunity. It soon became known that the novel coronavirus disease was evolving to be one that not only presents with respiratory symptoms but rather mild-to-severe systemic complications that took a toll on both patients and care providers. In this case, we highlight a rare instance of a coronavirus-induced syncope and fall; subsequent TBI with underlying hypoxia being a contributing factor further led to the discovery of systemic complications. This case aims to highlight the systemic complications COVID-19 is capable of while also raising awareness of the danger of hypoxia and low oxygen saturation with unknown additive effects by the novel coronavirus.

## References

[1] (2021). Centers for Disease Control and Prevention: symptoms of coronavirus. https://www.cdc.gov/coronavirus/2019-ncov/symptoms-testing/symptoms.html.

[2] Li J, Yu X, Hu S, Lin Z, Xiong N, Gao Y (2020). COVID-19 targets the right lung. Crit Care.

[3] Temgoua MN, Endomba FT, Nkeck JR, Kenfack GU, Tochie JN, Essouma M (2020). Coronavirus disease 2019 (COVID-19) as a multi-systemic disease and its impact in low- and middle-income countries (LMICs). SN Compr Clin Med.

[4] Guan WJ, Ni ZY, Hu Y (2020). Clinical characteristics of coronavirus disease 2019 in China. N Engl J Med.

[5] (2021). Centers for Disease Control and Prevention: key facts about Influenza (flu). https://www.cdc.gov/flu/about/keyfacts.htm.

[6] Demetriades D, Kuncir E, Velmahos GC, Rhee P, Alo K, Chan LS (2004). Outcome and prognostic factors in head injuries with an admission Glasgow Coma Scale score of 3. Arch Surg.

[7] Servadei F, Murray GD, Teasdale GM (2002). Traumatic subarachnoid hemorrhage: demographic and clinical study of 750 patients from the European brain injury consortium survey of head injuries. Neurosurgery.

[8] Mesfin FB, Gupta N, Shapshak AH, Taylor RS (2020). Diffuse axonal injury. https://www.ncbi.nlm.nih.gov/books/NBK448102/.

[9] Meythaler JM, Hazlewood J, DeVivo MJ, Rosner M (1998). Elevated liver enzymes after nontraumatic intracranial hemorrhages. Arch Phys Med Rehabil.

[10] Wang C, Zhang H, Cao X (2020). Red cell distribution width (RDW): a prognostic indicator of severe COVID-19. Ann Transl Med.

[11] Huang W, Berube J, McNamara M (2020). Lymphocyte subset counts in COVID-19 patients: a meta-analysis. Cytometry A.

[12] Schutgens RE (2020). D-dimer in COVID-19: a guide with pitfalls. Hemasphere.

[13] Navas-Blanco JR, Dudaryk R (2020). Management of respiratory distress syndrome due to COVID-19 infection. BMC Anesthesiol.

[14] Iadecola C, Anrather J, Kamel H (2020). Effects of COVID-19 on the nervous system. Cell.

[15] Horby P, Lim WS, Emberson JR (2021). Dexamethasone in hospitalized patients with Covid-19. N Engl J Med.

[16] Helms J, Tacquard C, Severac F (2020). High risk of thrombosis in patients with severe SARS-CoV-2 infection: a multicenter prospective cohort study. Intensive Care Med.

[17] Peckham H, de Gruijter NM, Raine C (2020). Male sex identified by global COVID-19 meta-analysis as a risk factor for death and ITU admission. Nat Commun.

